# The generation of a simian adenoviral vectored HCV vaccine encoding genetically conserved gene segments to target multiple HCV genotypes

**DOI:** 10.1016/j.vaccine.2017.10.079

**Published:** 2018-01-04

**Authors:** Annette von Delft, Timothy A. Donnison, José Lourenço, Claire Hutchings, Caitlin E. Mullarkey, Anthony Brown, Oliver G. Pybus, Paul Klenerman, Senthil Chinnakannan, Eleanor Barnes

**Affiliations:** a Peter Medawar Building and Translational Gastroenterology Unit, Nuffield Department of Medicine, University of Oxford, UK; b Department of Zoology, University of Oxford, UK

**Keywords:** T cell, HCV vaccine, Conserved segments, Cross-reactivity, Simian adenovirus

## Abstract

**Background:**

Hepatitis C virus (HCV) genomic variability is a major challenge to the generation of a prophylactic vaccine. We have previously shown that HCV specific T-cell responses induced by a potent T-cell vaccine encoding a single strain subtype-1b immunogen target epitopes dominant in natural infection. However, corresponding viral regions are highly variable at a population level, with a reduction in T-cell reactivity to these variants. We therefore designed and manufactured second generation simian adenovirus vaccines encoding genomic segments, conserved between viral genotypes and assessed these for immunogenicity.

**Methods:**

We developed a computer algorithm to identify HCV genomic regions that were conserved between viral subtypes. Conserved segments below a pre-defined diversity threshold spanning the entire HCV genome were combined to create novel immunogens (1000–1500 amino-acids), covering variation in HCV subtypes 1a and 1b, genotypes 1 and 3, and genotypes 1–6 inclusive. Simian adenoviral vaccine vectors (ChAdOx) encoding HCV conserved immunogens were constructed. Immunogenicity was evaluated in C57BL6 mice using panels of genotype-specific peptide pools in *ex-vivo* IFN-ϒ ELISpot and intracellular cytokine assays.

**Results:**

ChAdOx1 conserved segment HCV vaccines primed high-magnitude, broad, cross-reactive T-cell responses; the mean magnitude of total HCV specific T-cell responses was 1174 SFU/10^6^ splenocytes for ChAdOx1-GT1-6 in C57BL6 mice targeting multiple genomic regions, with mean responses of 935, 1474 and 1112 SFU/10^6^ against genotype 1a, 1b and 3a peptide panels, respectively. Functional assays demonstrated IFNg and TNFa production by vaccine-induced CD4 and CD8 T-cells. *In silico* analysis shows that conserved immunogens contain multiple epitopes, with many described in natural HCV infection, predicting immunogenicity in humans.

**Conclusions:**

Simian adenoviral vectored vaccines encoding genetic segments that are conserved between all major HCV genotypes contain multiple T-cell epitopes and are highly immunogenic in pre-clinical models. These studies pave the way for the assessment of multi-genotypic HCV T-cell vaccines in humans.

## Highlights

• This study describes HCV prophylactic vaccines designed to target multiple genotypes.
• HCV conserved immunogens were encoded within a simian adenoviral vector (ChAdOx1).
• Vaccines are highly immunogenic in mice and induce T-cells targeting multiple genotypes.
• HCV immunogens contain multiple human T-cell epitopes as defined in natural infection.

## 1 Introduction

Hepatitis C virus (HCV) infects approximately 170 million people worldwide and is a leading cause of end-stage liver disease. HCV treatment has become highly effective in the era of directly acting antivirals (DAA), with the latest drugs showing high efficacy against all HCV genotypes [1], [2]. However, the new drugs are expensive [3] and treatment rates remain low ranging from 3.5% in Europe to 21% in the US [4], [5]. Furthermore, successful treatment does not prevent re-infection, a particular problem in intra-venous drug using populations [6], [7]. Notably, a recent World Health Organization (WHO) report shows that even with effective treatment, available new HCV infections in 2015 exceeded the number of people dying of the disease or receiving curative therapy, leading to an increase in HCV prevalence [8]. Therefore, a HCV vaccine remains an important goal.

A major challenge for HCV vaccine development is the extensive viral variability of HCV that exists as seven major genotypes globally that are approximately 80% genetically homologous, and numerous HCV subtypes [9]. Whilst HCV genotype-1 is the most common genotype world wide, other genotypes are distributed in areas with high HCV prevalence with genotype-2 common in Asia, genotype-3 in the UK, Asia and former USSR, genotype-4, 5 and 7 in Africa, and genotype-6 in Asia [9]. In addition, HCV exists as a viral population of closely related genetic variants (quasispecies) within the infected host. Immunodominant T-cell responses in natural HCV infection are known to focus on a small number of epitopes that often show high sequence variability [10]. However, T-cell specificity is distinct between HCV genotypes [11] with limited T-cell cross-reactivity against common sequence variants at frequently detected T-cell targets [12].

We have previously shown that HCV vaccine strategies using simian adenovectors encoding HCV genotype-1b non-structural regions induce high magnitudes of HCV specific T-cells at epitopes dominant in natural infection [13], [14]. However, we observed reduced T-cell cross-reactivity of vaccine-induced T-cells against other genotypes [13] and against circulating viral variants at epitopes derived from the same genotype [15]. An effective global T-cell vaccine will need to generate T-cell responses capable of recognising viral variant epitopes between both quasispecies, and between people infected with different genotypes.

In this study, we introduce an immunogen design approach based on the selection of HCV genomic regions that are highly conserved between HCV genotypes with the exclusion of variable HCV epitopes [16]. We hypothesise that conserved HCV sequences carry a detrimental fitness cost if mutations occur in these regions. Therefore vaccine induced T-cells responses to conserved viral segments that are cross-reactive against multiple HCV genotypes will be capable of controlling and eliminating the virus during primary infection as viral mutation to escape T-cell recognition will carry a significant fitness cost to the virus.

We used world-wide HCV prevalence data [9] to inform the rational design of three distinct HCV immunogens targeting (i) genotype-1 alone - the most prevalent genotype globally (GT1), (ii) genotypes-1 and 3 - the two most commonly infecting genotypes in the UK [17] and world wide (GT1/3); and (iii) genotypes-1 to 6 - covering all common HCV genotypes globally (GT1-6) [18]. Novel conserved immunogens were encoded in a simian adenoviral vector ChAdOx1 [19], known to be capable of inducing high magnitude T-cell responses, which also circumvents the issue of pre-existing immunity to adenoviral vectors that may limit vaccine efficacy.

We assessed designed antigens for immunogenicity using HLA epitope prediction programs, through the analysis of epitopes described in natural HCV infection and finally through evaluating vaccine immunogenicity *in vivo* in pre-clinical mouse studies.

## 2 Methods

### 2.1 The selection of HCV sequences

Full-length genotype HCV sequences from the HCV sequence database www.hcv.lanl.gov, and in-house genotype 3 sequences were used for immunogen design (n = 216, Supplementary Table S1). Three different sequence datasets were generated: (a) HCV genotype-1 (GT1) n = 96, (b) HCV genotypes 1 and 3 (GT1/3) n = 72 and (c) HCV genotypes 1–6 (GT1-6) n = 216. Sequences from different research groups and countries were selected for each strain and manually checked for human origin. HCV subtypes were chosen according to common circulating viral strains [18]. Genotype-7 was not included as only one sequence has been published [18].

### 2.2 Definition of conserved HCV viral segments

Sequence diversity (normalized raw diversity, NDR) was calculated using pairwise Hamming distance. A sliding window (W) of 20 amino-acid (AA) starting at position zero and advancing one AA at a time was used to measure Hamming distance between all pairs of sequences in the alignment. A window size of 20 AA was selected to contain potential CD4 and CD8 epitopes (8–12 AA and 11–16 AA respectively (Supplementary Fig. S1). To define conserved segments in the dataset, we selected a threshold equal to the lowest quartile (denoted Θ) of the overall diversity distribution.

### 2.3 Selection of a circulating isolate for final immunogen design

A consensus sequence was generated for each dataset (GT1, GT1/3 and GT1-6); hereafter named “overall consensus”, and all subtypes (e.g. for genotype-1 and genotype-3 in the GT1/3 analysis); hereafter named “subtype consensus”. A circulating HCV isolate from a subtype within the dataset, with a highest homology to the overall consensus was selected and included in conserved immunogens.

### 2.4 Prediction analysis for T- and B-cell epitopes and proteasomal cleavage sites

Prediction of proteasomal cleavage sites was run using server NetChop 3.1, (version C-term 3.0). Epitope prediction analysis was run using online epitope prediction programs NetMHC, Syfpeithi and BIMAS with a cut-off for strong epitope binders of <0.5, >20, and >100, respectively. Potential immunogenicity of HCV immunogens was evaluated using the NetMHC prediction server (v3.4) for T-cell epitopes. B-cell epitopes were predicted with BepiPred 2.0 using a cut-off for strong binding epitopes 0.55 and an epitope prediction length of 5–20 AAs. Junctions between two concatenated conserved segments were assessed for strong binders using two independent prediction algorithms for HLA class-I and class-II types at HLA population frequency over 2% (NCBI database). Predicted strong binders were abrogated using linkers consisting of 2–6 AA (glycine/proline or glycine/serine combinations) as previously described [20], [21]. A blast analysis was performed (https://blast.ncbi.nlm.nih.gov) to ensure there was no homology with the human genome at junctional regions.

### 2.5 Analysis of HCV T-cell epitopes in natural infection

T-cell epitopes/targets were mapped along the HCV genome to assess the association of these with genomic variability. HCV genotype-1 and genotype-3 epitopes were obtained from the immune epitope database resource (IEDB) and from those experimentally defined in our laboratory [12]. Epitopes were crosschecked with primary publications and duplications, epitope variants and epitopes described in non-human organisms were excluded.

### 2.6 Vaccine production

Conserved HCV immunogens were synthetically produced using “humanized” amino acid codons (GeneArt, ThermoFisher Scientific) [22] and cloned into a pENTR4 vector [19], [23]. Coding cassettes within the pENTR4 vector were cloned into ChAdOx1 plasmids (Thermo Fisher Scientific LR gateway cloning procedure) and then linearized with PmeI and transfected into T-REx™-293 cells (Thermo Fisher Scientific) for generation of viral vector vaccines. ChAdOx1 HCV vaccines were manufactured by the Viral Vector Core Facility (Jenner Institute, University of Oxford, Oxford, UK) [23]. Immunogen expression was evaluated in HEK 293a cells by Western blot using anti-HCV core 1b antibody (C7-50, AB2740, Abcam); this detects AA sequence 21–40 (DVKFPGVGQIVGGVYLLPR) of HCV core and is contained within all HCV immunogens”.

### 2.7 Animals

Experiments were performed at the Biomedical Services Building, Oxford according to UK Home Office Regulations (licence numbers 30/2744 and 30/3293) and approved by the local ethical review board at the University of Oxford. All animal experiments complied with the ARRIVE guidelines and were carried out in accordance with the U.K. Animals (Scientific Procedure) Act, 1986. Three groups of four age and sex matched C57BL6 mice were vaccinated intramuscularly (dose of 1 × 10^8^ infectious units (I.U.) in 40 l per mouse) at 4–6 weeks of age with ChAdOx1 vaccine containing the long version of gt1-6 immunogen (ChAdOx1-gt1-6L-TPA-LS). Mice were sacrificed 2 weeks post-immunization.

### *2.8* *Ex vivo* interferon-(IFN)-γ-linked ELISpot assays

Splenocytes were isolated from harvested spleens and evaluated in *ex vivo* interferon-(IFN)-γ-linked ELISpot assays as previously described [12], [13]. In brief, pre-coated ELISpot plates (with anti-IFNγ monoclonal antibody (0.5 μg/well, Mabtech) were blocked with R10 (RPMI Sigma, 10% FCS, Penicillin and Streptomycin added). 200,000 PBMCs/well were stimulated for 18 h with HCV peptide sets (3 μg/ml), DMSO and concanavalin A (10 μg, Sigma) serving as negative and positive controls, in duplicates for each condition. All ELISpot assays were strongly positive for concanavalin A. HCV genotype-1a (H77), genotype-1b (J4b) and genotype-3 (K3a) peptides of 15–18 AA in length overlapping by 11 AA spanning the whole HCV genome and grouped into pools representing HCV viral proteins (e.g. core, E1, E2, NS2) were used. Spot-forming units (SFU) were counted on an automated ELISpot plate reader. Positive responses were determined by calculating (mean SFU/10^6^PBMC in test wells - negative control wells)+ 3× SD. Mann-Whitney tests (for non-Gaussian distributions) were used to determine statistical differences of the means between each HCV genotype stimulus and the DMSO control.

### 2.9 Intracellular staining assays

Mouse splenocytes at 1 × 10^6^ cells/100 μL were stimulated with HCV genotype-1a and -3a peptide pools covering all HCV proteins at 2 μg/ml or PMA (phorbol 12-myristate 13-acetate)/ionomycin (50 and 500 ng/mL respectively), or unstimulated (DMSO; 3 ng/mL). BD GolgiPlug™ was added (4 μl/mL) 1 h later, cells incubated overnight (37 C), stained with fixable-NIR live/dead dye (Life Technologies, Carlsbad, CA, USA) at 1:1000 dilution, and CD3-eflour 450, CD8-peridinin chlorophyll protein (perCp) Cy5.5, and CD4-AlexFlour700 (all 1:50) antibodies. Cells were fixed in 1% formaldehyde for 20 min at 4 °C, and re-suspended in PBS for overnight storage at 4 °C. Cell permeabilization was performed the next day using BD Perm/Wash™ Buffer (Fix/Perm kit, BD Biosciences, San Jose, CA, USA) followed by intracellular staining using IFNγ-PE (phycoerythrin) and TNFα-FITC (1:50). All flow cytometry was performed on a BD LSRII machine and analysed using FlowJo vX.0.7 (Treestar, Ashland, OR, USA).

## 3 Results

### 3.1 Definition of HCV conserved segments with significant overlap between genotypes

**Fig. 1:**
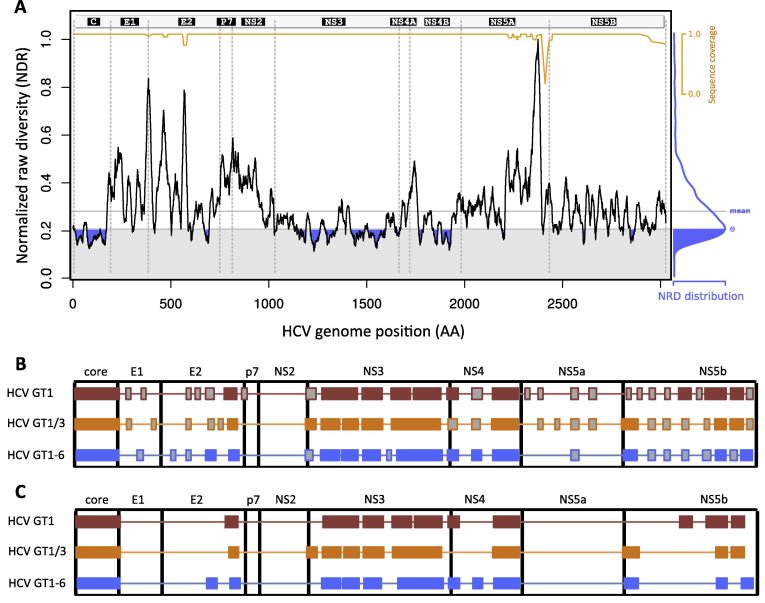
Sequence diversity plot of the full HCV genome with defined conserved HCV segments. (A) Sequence diversity (normalized raw diversity, NDR) for an example sequence dataset is shown for the full HCV genome (sequence dataset HCV GT1/3a, containing 72 sequences) using a window size of W = 20. For vaccine design, segments with variability below 25% of the overall diversity distribution (first quartile Θ, marked blue) were defined as conserved and selected for conserved immunogens. For visualization purposes, diversity obtained from hamming distance measures (NDR) is normalized to 1 using the maximum found (black full line). Gaps in the dataset were ignored in the calculation of NDR, and we therefore present the sequence coverage used for the NDR measurement at each window (orange full line). Boundaries of genome regions are marked by vertical dashed lines, with gene nomenclature on the top. (B) Schematics of long and (C) short versions of conserved HCV immunogens for HCV GT1 (red), GT1/3 (orange) and GT1-6 (blue) are depicted.

HCV sequence diversity was calculated for GT1, GT1/3 and GT1-6 sequence datasets, and conserved viral segments with 95.5% (GT1), 94.7% (GT1/3) and 90.2% (GT1-6) homology were obtained using a cut-off at 25% of total variability within each dataset (Fig. 1a). Conserved segments were defined within each dataset and these were concatenated to form “long” immunogens (Fig. 1b): 30 for GT1 (total 1543 AA), 27 for GT1/3 (total 1443 AA) and 24 for GT1-6 (total 1350 AA). Significant overlap of conserved segments was observed at 1141 AA positions; with 1043 (91.4%) homologous AAs between conserved consensus sequences defined for GT1, GT1/3 and GT1-6 (Supplementary Fig. S2). As vaccines encoding immunogens over 1000 AA may be difficult to manufacture, we designed additional shorter immunogens by ranking conserved sequence segments by length and selecting the longest segments up to a total of ∼ 1000 AA, with resulting immunogen lengths of 1043, 1010 and 1041 AA for HCV GT1, GT1/3 and GT1-6, respectively (subsequently termed “short immunogens”/‘S’, e.g. Gt1-6S) (Fig. 1c).

### 3.2 Conserved sequence segments are highly homologous between genotypes

**Fig. 2:**
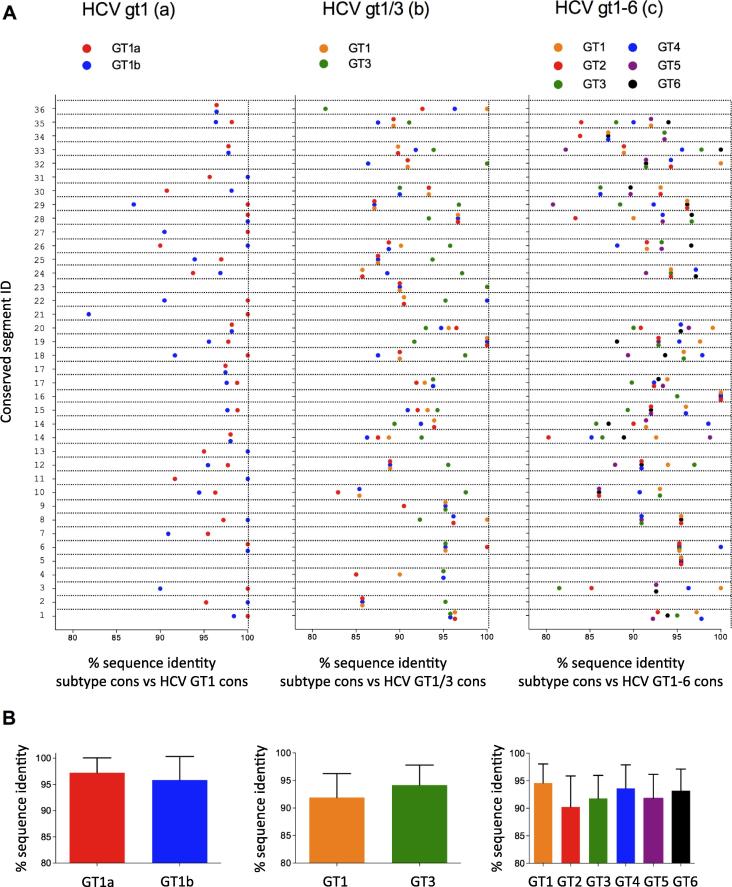
Patient sequence selection for final immunogen design. (A) Similarity of subtype consensus sequences (depicted as coloured spots) to overall consensus sequences at each conserved segment, shown for analyses HCV GT1 (a, left), HCV GT1/3 (b, middle) and HCV GT1-6 (c, right) immunogens. Conserved segment ID numbers (as defined in Supplementary Fig. S2) are marked on the y-axis. (B) Overall similarity between subtype consensus sequences and overall consensus sequences for all conserved segments of analyses HCV GT1 (left), HCV GT1/3 (middle) and HCV GT1-6 (right). Cons: consensus.

Next, we determined sequence homology between genotypes at defined conserved segments. Over 80% sequence homology was observed at all defined conserved segments between subtype and overall consensus sequences (Fig. 2a). Overall, high mean similarities were observed between subtype consensuses and overall consensuses; 97.2% (GT1a) and 95.8% (GT1b) for the GT1 analyses, 91.9% (GT1) and 94.1% (GT3) for the GT1/3 analyses, and 90.2% (GT2) to 94.6% (GT1) for the GT1-6 analyses (Fig. 2b). Natural HCV isolates optimally matched to the overall consensus were used in the final immunogens (Supplementary Table S2), as using consensus sequences differing from natural HCV isolates may lead to T-cells that target non-HCV epitopes.

### 3.3 Identifying proteasomal cleavage sites, T- and B-cell epitopes within conserved immunogens

*Using computer algorithms:* Using an online prediction algorithm (http://www.cbs.dtu.dk/NetChop), between 357 and 374 proteasomal cleavage sites were predicted for short conserved immunogens, with 462 and 545 cleavage sites predicted for long immunogens (Supplementary Table S3). The potential immunogenicity of conserved immunogens was evaluated using an online epitope prediction algorithm (http://www.cbs.dtu.dk/NetMHC) shown to predict MHC class-I epitopes with high accuracy [24], [25]. Between 176 and 215 strong binding MHC class-I epitopes were predicted for the short immunogens, and 251–318 for the longer immunogens (Supplementary Fig. S3a). The number of predicted strong binders was associated with immunogen length, and conserved segments were well populated with predicted CD8 epitopes of a wide range of different HLA types (Supplementary Fig. S3b and c). Using BepiPred-2.0 (http://www.cbs.dtu.dk/services/BepiPred-2.0/) [26] between 17 and 19 strong binding B-cell epitopes were predicted for short immunogens, and 18–23 for long immunogens (Supplementary Table S4). Of note, two E2 antigenic sites now considered prime targets for B-cell vaccine design (AS412 and AS434) [27] are not included in the designed conserved immunogens.

**Fig. 3:**
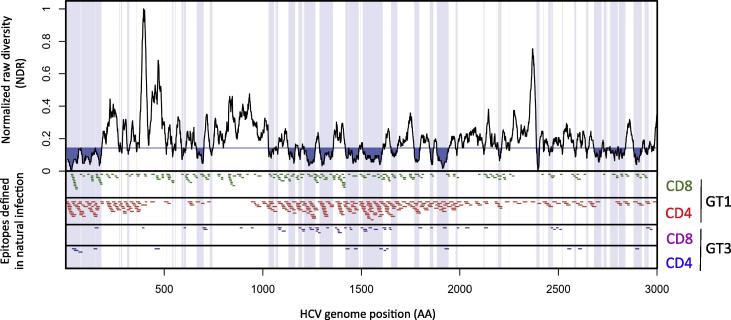
Epitopes defined in natural HCV genotype-1 and genotype-3 infection populate HCV conserved and variable regions. HCV sequence heterogeneity (top panel, normalized raw diversity [NDR]) as defined for HCV GT1-6 with conserved regions marked blue and HCV epitopes described in natural HCV infection (bottom panels) are shown. T-cell epitopes described in natural HCV infection are depicted for HCV genotype-1 CD8 (green) and CD4 (red) epitopes, and HCV genotype-3 CD8 (purple) and CD4 (blue) epitopes.

*T-cell epitopes defined in natural infection:* Next, we determined whether CD4 and CD8 restricted epitopes described in natural infection were detected in conserved viral segments; 89 of 215 CD8 (41.4%) and 170 of 320 CD4 (53%) of HCV genotype-1 epitopes described in natural infection mapped to conserved viral segments within the HCV genotype 1 dataset (Fig. 3). HCV genotype-1 CD4 T-cell epitopes mapped to conserved rather than variable viral segments (p < .0001), whereas CD8 epitopes were equally distributed across the viral genome (Supplementary Fig. S4). Similar results albeit at lower numbers were observed when analysing HCV genotype-3 specific epitopes, with 17 of 48 CD8 (35.4%) and 9 of 14 CD4 (64.3%) HCV genotype-3 specific epitopes mapping to conserved viral segments within the HCV GT1/3 dataset (Fig. 3). T-cell epitopes for HCV genotypes 2, 4, 5 and 6 have rarely been evaluated experimentally using genotype specific peptides and were therefore not included in our analysis.

We also assessed whether epitopes linked to spontaneous resolution in natural HCV infection were included in the conserved immunogens [28], [29], [30]. Of four epitopes associated with resolution in HCV genotype-1 infection, one (E2_541_NTRPPLGNW) was included in the HCV GT1 construct, whilst NS5B_2629_KSKKTPMGF is contained in the HCV GT1/3a and HCV GT1-6 constructs (but differs by one AA; NS5B_2629_**T**SKKTPMGF). A protective epitope at position NS5B_2841_ has been described in genotype-1 infection and is contained in the HCV GT1/3a and HCV GT1-6 constructs, but as a variant (NS5B_2841_**V**RM**V**LMTHF) that is non-immunogenic (Table 1) [30].

**Table 1:** Position of HCV epitopes linked to protection in HCV genotype 1 regarding defined conserved viral segments. HCV genotype-1 epitopes associated with spontaneous resolution (SR), as described in the literature (reference given; Ref), are shown alongside epitope variants that associated with immune escape. The HLA restriction of the epitope is shown. The position of the epitope within the HCV protein (and amino-acid position relative to HCV H77) is given. The vaccine constructs that contain the epitope are defined alongside the epitope variant contained within each vaccine construct. (HLA = human leukocyte antibody, AA = amino acid).

Epitope associated with SR	HLA restriction	Position in HCV genome	Ref.	Vaccine construct	Included in vaccine	Epitope variant in vaccine
NTRPPLGNW Escape variant: NTRPPXGNW	B*57	E2_541_	[28]	GT1	yes	NTRPPLGNW
				GT1/3a	no	
				GT1-6	no	

TVYHGAGTK Escape variant: TVYHGAGXX	A*03	NS3_1080_	[29]	GT1	partially	TVYHGAG
				GT1/3a	no	
				GT1-6	no	

KSKKTPMGF Escape variant: XXKKXPMGF	B*57	NS5B_2629_	[28]	GT1	partially	PMGF
				GT1/3a	yes	TSKKTPMGF
				GT1-6	partially	KKTPMGF

ARMILMTHF Escape variant: XRMILXTHF	B*27	NS5B_2841_	[30]	GT1	partially	ARMIL
				GT1/3a	partially	VRMVL
				GT1-6	yes	VRMVLMTHF

### 3.4 T-cell epitopes in junction regions and abrogation through linker design

As conserved HCV immunogens are concatenated from multiple sequence segments, T-cell responses may be generated to artificial non-HCV regions between adjacent conserved segments. The immunogenicity of junction regions was evaluated using online epitope prediction servers (NetCTL, BIMAS, Sythpeithi) [31], [32]. Approximately 15% of all predicted strong binding T-cell epitopes were located in junction areas between conserved segments. To avoid priming of T-cell responses against artificial epitopes, those predicted by two prediction servers were abrogated through the insertion of glycine/proline [21] or glycine/serine [20] AA linkers (Supplementary Fig. S5). As identical linkers may lead to dimerization during protein expression a variety of linkers was used. Junction regions were then reassessed, showing a reduction of strong predicted class-I binders in junction areas by 39% (GT1), 41% (GT1/3) and 24% (GT1-6) for the long conserved immunogens, respectively (Supplementary Fig. S5).

### 3.5 Conserved segment vaccines are highly immunogenic in mice

**Fig. 4:**
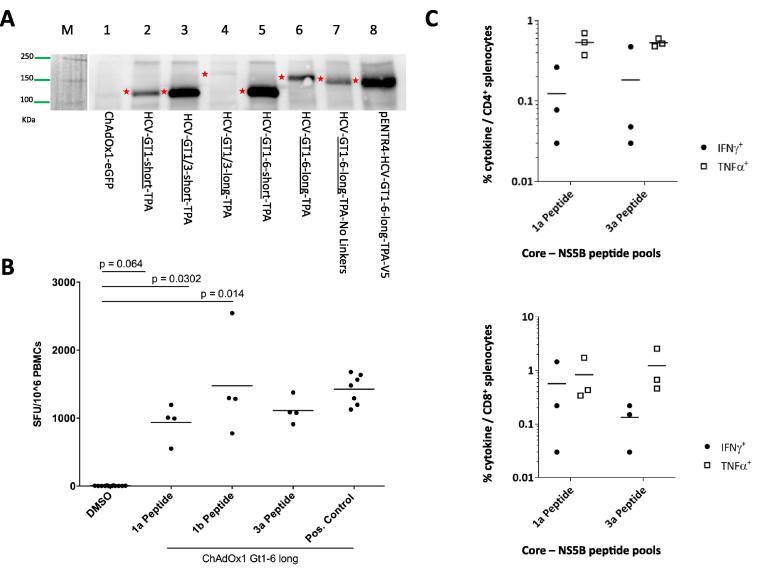
Production of HCV conserved immunogens and pre-clinical assessment in mice. (A) Core expression analysis from ChAdOx1 based vaccines encoding 6 different conserved HCV immunogens in HEK293A cells at a MOI of 100 after an infection duration of 24 h. Controls included a ChAdOx1 vector and a pENTR4-HCV-GT1-6-long-TPA construct. M – Marker Precision Plus Dual Color (BioRad). (B) Magnitude of murine splenocyte-derived T-cell responses measured in IFNγ-ELISpot assays using HCV genotype-1a, genotype-1b and genotype-3a peptide pools in C57BL6 mice vaccinated with the viral vector ChAdOx1 containing the long construct of conserved HCV GT1-6 immunogen (ChAdOx1-gt1-6L-TPA) 2 weeks after immunization at dose 1 × 10^8^ I.U. given intramuscularly. Spot-forming units (SFU) were normalized to 10^6^ cells. Results were defined as significant when p < .05 when compared with the DMSO background (Mann-Whitney test). (C and D) Total magnitude of C57BL6 splenocyte-derived CD4 (C) and CD8 (D) T-cell responses measured in intracellular staining assays and flow cytometry analysis. IFNγ^+^ and TNFα^+^,cytokine production following stimulation with core-E1-E2, NS3-4, and NS5 peptide pools for genotype-1a and -3a was assessed. Mice were sacrificed 2-weeks after immunization with ChAdOx1-gt1-6L-TPA at dose 1 × 10^8^ I.U. given intramuscularly (I.M.).

Final constructs included linkers to abrogate artificial epitopes, a Kozak sequence to maximize protein expression [33] and the tissue plasminogen activator leader sequence (TPA-LS) to optimize peptide presentation (Supplementary Table S2) [36], and were encoded in the simian adenovirus ChAdOx1 [19]. Immunogen expression was demonstrated by Western blot using the monoclonal antibody against HCV core in infected human embryonic kidney cells (Fig. 4a) [34]. HCV GT1-6-TPA showed high levels of expression and was further assessed in C57BL6 mice. High magnitude T-cell responses against HCV peptides from subtypes 1a (mean 935 SFU/10^6^), 1b (mean 1474 SFU/10^6^) and 3a (mean 1112 SFU/10^6^) were demonstrated (Fig. 4b). When assessed using intracellular cytokine assay and flow cytometric analysis vaccine-induced CD4 and CD8 T-cells produce IFNγ and TNFα on stimulation with HCV genotype 1 and 3 peptides (Fig. 4c and d), indicating that designed conserved HCV immunogens are immunogenic and capable of generating cross-reactive immune responses [11], [12].

## 4 Discussion

One of the key obstacles in generating an effective vaccine for HCV is the extensive variability of the HCV genome and the geographical distribution of genetically distinct viral genotypes globally [35]. We have addressed this through the design and generation of a T-cell vaccine using a simian adenoviral vector encoding immunogens from HCV regions that are conserved between viral genotypes. The vaccines generated are predicated to contain multiple HCV human epitopes that are cross-reactive between major genotypes in a pre-clinical model.

Sequence datasets generated for immunogen design used full-length HCV sequences only; this approach ensured equal coverage of conserved and variable viral regions, since variable viral regions are more difficult to sequence and underrepresented in sequence databases. We showed that conserved segments contained protein sequence that was more than 91.4% homologous between genotypes and are expected therefore to prime HCV specific T-cell responses that are highly cross-reactive between genotypes. Ultimately, we used naturally circulating HCV isolates rather than consensus sequences in the immunogen design since consensus sequences may contain both artificial epitopes (not found in the wild) and dominant escape mutations at epitopes where the restricting HLA type is common.

One issue with an approach that uses conserved segments in immunogen design is the possibility that there is a lack of immunological pressure driving viral mutations associated with genetic conservation in these regions [36], [37]. We therefore assessed whether T- and B-cell epitopes could be found in these regions through *in silico* analysis using online prediction programs and through an evaluation of HCV specific T-cell epitopes described in natural infection. *In silico* epitope prediction confirmed the presence of multiple proteasomal cleavage sites and high numbers of strong T- and B-cell binders in conserved regions suggesting that these are able to induce immune responses. Whilst we recognise that *in silico* algorithmic epitope prediction only remodels a part of the complex epitope presentation pathway (peptide binding to MHC complexes), this is the most selective step of the epitope presentation pathway and contributes to about 90% of immunodominance [10].

We also show that epitopes described in natural infection are contained within conserved regions indicating that these have the potential to prime HCV specific response in humans *in vivo*. This result is in keeping with a published analysis of HCV epitopes deposited on the IEDB showing that frequently recognized T-cells epitopes correlate with low sequence variability [38]. In addition, we show here that if HCV genotype-1-specific CD4 and CD8 T-cell targets are analysed separately, CD4 epitopes preferentially map to conserved regions, whereas CD8 epitopes are equally distributed throughout conserved and variable viral segments. This is in line with the observation that viral escape at CD4 T-cell epitopes is relatively unusual [39], which may contribute to the central role that CD4 cells play in spontaneous resolution of acute HCV infection [40], [41]. Notably, immune-dominant but highly variable CD8 epitopes such as NS3_1073_CINGVCWTV and NS3_1406_KLVALGINAV are not included in our HCV conserved immunogens.

Some T-cell epitopes previously linked to the spontaneous resolution of HCV infection were also excluded from our immunogen. However, we predict that excluding these may potentially lead to the “up-ranking” of sub-dominant epitopes, as shown for a conserved HIV vaccine, where serially deleting immunodominant epitopes tripled the frequencies of immune responses to previously subdominant epitopes [42]. Furthermore, strong T-cell responses to conserved regions subdominant in natural infection were readily detected when evaluating conserved vaccines for other highly variable viruses such as HIV [34], [43], and dengue [44], and high magnitudes of HIV-specific T-cells inhibiting HIV replication were detected in mice [34], [45] macaques [46], and in a Phase I clinical trial using a conserved segment vaccine approach [43]. Although the E2 antigenic sites (AS412 and AS434) that are now considered prime targets for B-cell vaccine design were not included in HCV conserved immunogens [27], it may be prudent to combine vaccine platform technologies with the aim of inducing both B- and T-cell responses.

Although *in silico* analysis and the assessment of T-cell epitopes in natural infection predict that the conserved segment HCV vaccines will generate HCV specific immunity, concatenating conserved fragments into a chimeric protein may impact on protein processing and presentation, and therefore on vaccine-induced T-cell responses. It was therefore important to show that HCV conserved immunogens are processed and capable of priming immune responses *in vivo*. We show that HCV conserved immunogens encoded in a simian adenoviral vector (ChAdOx1) induce strong HCV-specific T-cell responses that produce IFN-γ and TNF-α in mice and are cross-reactive with multiple HCV subtype antigens. A limitation of this study is that epitope presentation and T-cell specificity in mice is based on H-2Dd molecules and therefore distinct from human HLA-based human epitope presentation. To properly address this vaccines will need to be assessed humanized pre-clinical models and humans.

Alternative vaccine developmental strategies include those encoding single HCV isolates or epitopes [47]. A clear disadvantage with these approaches is that these may have limited applicability in a global strategy where human populations are HLA diverse, and multiple HCV genotypes circulate. Other approaches that specifically aim to induce broad, cross-reactive HCV-specific T-cell responses include the selection of single conserved T-cell epitopes [48], [49], ancestral sequences designed to minimise differences between the immunogen and circulating viruses [50] or recombinant mosaic vaccines that encode computationally generated immunogens through machine learning; these are designed to closely resemble natural proteins to ensure optimal antigen processing and maximize T-cell coverage [51], [52]. This approach has shown some promise in animal models of HIV [53], [54] and pre-clinical studies of HCV [55] although T-cells induced by mosaic vaccines may still preferentially target variable viral regions.

In summary, we have developed HCV simian adenoviral vectored vaccines specifically designed to target all HCV genotypes by encoding HCV conserved genomic segments from all major HCV genotypes. We show through *in silico* analysis that conserved regions are highly populated with epitopes predicted by online epitope prediction servers and by those described in natural infection. These vaccines generate high magnitude HCV-specific T-cell responses that cross-react with multiple HCV subtypes in a pre-clinical mouse model. This study paves the way for the assessment of an HCV T-cell vaccine in with the potential to target multiple HCV genotypes.

## References

[1] Foster G.R. (2015). Sofosbuvir and velpatasvir for HCV genotype 2 and 3 infection. N Engl J Med.

[2] Feld J.J. (2015). Sofosbuvir and velpatasvir for HCV genotype 1, 2, 4, 5, and 6 infection. N Engl J Med.

[3] Cammà C. (2013). Cost-effectiveness of boceprevir or telaprevir for previously treated patients with genotype 1 chronic hepatitis C. J Hepatol.

[4] Volk M.L. (2010). Antiviral therapy for hepatitis C: why are so few patients being treated?. J Antimicrob Chemother.

[5] Razavi H., Estes C., Pasini K., Gower E., Hindman S. (2013). HCV treatment rate in select european countries in 2004–2010. J Hepatol **Supplement**.

[6] Franco S. (2014). Detection of a sexually transmitted Hepatitis C virus protease inhibitor-resistance variant in a human immunodeficiency virus-infected homosexual man. Gastroenterology.

[7] Sulkowski M. (2014). Daclatasvir plus sofosbuvir for previously treated or untreated chronic HCV infection. N Engl J Med.

[8] WHO. WHO | Global hepatitis report. WHO (2017). http://www.who.int/hepatitis/publications/global-hepatitis-report2017/en/; 2017 [accessed 23.09.17].

[9] Messina J.P. (2014). Global distribution and prevalence of hepatitis C virus genotypes. Hepatology.

[10] Yewdell J.W. (2006). Confronting complexity: real-world immunodominance in antiviral CD8+ T cell responses. Immunity.

[11] Giugliano S. (2009). Degree of cross-genotype reactivity of hepatitis C virus-specific CD8+ T cells directed against NS3. Hepatology.

[12] von Delft A. (2015). The broad assessment of HCV genotypes 1 and 3 antigenic targets reveals limited cross-reactivity with implications for vaccine design. Gut.

[13] Barnes E, et al. Novel adenovirus-based vaccines induce broad and sustained T cell responses to HCV in man. Sci Transl Med 2012; 4: 115ra1. 10.1126/scitranslmed.3003155 PMC3627207 22218690

[14] Swadling L, et al. A human vaccine strategy based on chimpanzee adenoviral and MVA vectors that primes, boosts, and sustains functional HCV-specific T cell memory. Sci Transl Med 2014; 6: 261ra153. 10.1126/scitranslmed.3009185 PMC4669853 25378645

[15] Kelly C. (2014). Cross-reactivity of hepatitis C virus-specific vaccine induced T cells at immunodominant epitopes. Eur J Immunol.

[16] Roohvand F., Kossari N. (2012). Advances in hepatitis C virus vaccines, part two: advances in hepatitis C virus vaccine formulations and modalities. Expert Opin Ther Pat.

[17] Health Protection Agency. Hepatitis C in the UK: 2013 report. http://www.hpa.org.uk/Publications/InfectiousDiseases/BloodBorneInfections/HepatitisCInTheUK/1307HepatitisCintheUK2013report/; 2013 [accessed 10.03.14].

[18] Smith D.B. (2014). Expanded classification of hepatitis C virus into 7 genotypes and 67 subtypes: updated criteria and genotype assignment Web resource. Hepatology.

[19] Antrobus R.D. (2014). Clinical assessment of a novel recombinant simian adenovirus ChAdOx1 as a vectored vaccine expressing conserved Influenza A antigens. Mol Ther.

[20] Gilbert S.C. (1997). A protein particle vaccine containing multiple malaria epitopes. Nat Biotech.

[21] Berthoud T.K. (2011). Potent CD8+ T-cell immunogenicity in humans of a novel heterosubtypic influenza A vaccine, MVA−NP+M1. Clin Infect Dis.

[22] André S. (1998). Increased immune response elicited by DNA vaccination with a synthetic gp120 sequence with optimized codon usage. J Virol.

[23] Dicks M.D.J. (2012). A novel chimpanzee adenovirus vector with low human seroprevalence: improved systems for vector derivation and comparative immunogenicity. PLoS ONE.

[24] Lin H.H., Ray S., Tongchusak S., Reinherz E.L., Brusic V. (2008). Evaluation of MHC class I peptide binding prediction servers: applications for vaccine research. BMC Immunol.

[25] Lin H.H., Zhang G.L., Tongchusak S., Reinherz E.L., Brusic V. (2008). Evaluation of MHC-II peptide binding prediction servers: applications for vaccine research. BMC Bioinform.

[26] Jespersen MC, Peters B, Nielsen M, Marcatili P. BepiPred-2.0: improving sequence-based B-cell epitope prediction using conformational epitopes. Nucleic Acids Res 2017; 45: W24–W29. 10.1093/nar/gkx346 PMC5570230 28472356

[27] Kong L., Jackson K.N., Wilson I.A., Law M. (2015). Capitalizing on knowledge of hepatitis C virus neutralizing epitopes for rational vaccine design. Curr Opin Virol.

[28] Kim A.Y. (2010). Spontaneous control of HCV is associated with the expression of HLA-B*57 and preservation of targeted epitopes. Gastroenterology.

[29] Fitzmaurice K. (2011). Molecular footprints reveal the impact of the protective HLA-A*03 allele in hepatitis C virus infection. Gut.

[30] Neumann-Haefelin C. (2010). Protective effect of human leukocyte antigen B27 in hepatitis C virus infection requires the presence of a genotype-specific immunodominant CD8+ T-cell epitope. Hepatology.

[31] Rammensee H., Bachmann J., Emmerich N.P., Bachor O.A., Stevanović S. (1999). SYFPEITHI: database for MHC ligands and peptide motifs. Immunogenetics.

[32] Nielsen M. (2008). Quantitative predictions of peptide binding to any HLA-DR molecule of known sequence: NetMHCIIpan. PLoS Comput Biol.

[33] Kozak M. (1987). An analysis of 5’-noncoding sequences from 699 vertebrate messenger RNAs. Nucl Acids Res.

[34] Létourneau S. (2007). Design and pre-clinical evaluation of a universal HIV-1 vaccine. PLoS ONE.

[35] Smith D.B. (2013). Expanded classification of hepatitis C Virus into 7 genotypes and 67 Subtypes: updated criteria and assignment web resource. Hepatology.

[36] Ferguson A.L. (2013). Translating HIV sequences into quantitative fitness landscapes predicts viral vulnerabilities for rational immunogen design. Immunity.

[37] Carlson J.M., Le A.Q., Shahid A., Brumme Z.L. (2015). HIV-1 adaptation to HLA: a window into virus-host immune interactions. Trends Microbiol.

[38] Kim Y. (2012). A meta-analysis of the existing knowledge of immunoreactivity against hepatitis C virus (HCV). PLoS ONE.

[39] Fuller M.J. (2010). Selection-driven immune escape is not a significant factor in the failure of CD4 T cell responses in persistent hepatitis C virus infection. Hepatology.

[40] Day C.L. (2002). Broad specificity of virus-specific CD4+ T-Helper-cell responses in resolved hepatitis C virus infection. J Virol.

[41] Grakoui A. (2003). HCV persistence and immune evasion in the absence of memory T cell help. Science.

[42] Im E.-J. (2011). Protective efficacy of serially up-ranked subdominant CD8+ T cell epitopes against virus challenges. PLoS Pathog.

[43] Borthwick N. (2013). Vaccine-elicited human T cells recognizing conserved protein regions inhibit HIV-1. Mol Ther.

[44] Khan A.M. (2006). A systematic bioinformatics approach for selection of epitope-based vaccine targets. CellImmunol.

[45] Ondondo B., Brennan C., Nicosia A., Crome S.J., Hanke T. (2013). Absence of systemic toxicity changes following intramuscular administration of novel pSG2.HIVconsv DNA, ChAdV63.HIVconsv and MVA.HIVconsv vaccines to BALB/c mice. Vaccine.

[46] Koopman G. (2013). DNA/long peptide vaccination against conserved regions of SIV induces partial protection against SIVmac251 challenge. AIDS.

[47] Firbas C. (2006). Immunogenicity and safety of a novel therapeutic hepatitis C virus (HCV) peptide vaccine: a randomized, placebo controlled trial for dose optimization in 128 healthy subjects. Vaccine.

[48] Memarnejadian A., Roohvand F., Arashkia A., Rafati S., Shokrgozar M.A. (2009). Polytope DNA vaccine development against hepatitis C virus: a streamlined approach from in silico design to in vitro and primary in vivo analyses in BALB/c mice. Protein Pept Lett.

[49] Arashkia A., Roohvand F., Memarnejadian A., Aghasadeghi M.R., Rafati S. (2010). Construction of HCV-polytope vaccine candidates harbouring immune-enhancer sequences and primary evaluation of their immunogenicity in BALB/c mice. Virus Genes.

[50] Burke K.P. (2012). Immunogenicity and cross-reactivity of a representative ancestral sequence in hepatitis C virus infection. J Immunol.

[51] Fischer W. (2007). Polyvalent vaccines for optimal coverage of potential T-cell epitopes in global HIV-1 variants. Nat Med.

[52] Korber B.T., Letvin N.L., Haynes B.F. (2009). T-cell vaccine strategies for human immunodeficiency virus, the virus with a thousand faces. J Virol.

[53] Barouch D.H. (2010). Mosaic HIV-1 vaccines expand the breadth and depth of cellular immune responses in rhesus monkeys. Nat Med.

[54] Abdul-Jawad S. (2015). Increased valency of conserved-mosaic vaccines enhances the breadth and depth of epitope recognition. Mol Ther.

[55] Yusim K. (2013). Hepatitis C genotype 1 mosaic vaccines are immunogenic in mice and induce stronger T-cell responses than natural strains. Clin Vaccine Immunol.

