# Supplementary material for: The generation of a simian adenoviral vectored HCV vaccine encoding genetically conserved gene segments to target multiple HCV genotypes
Source: Vaccine. 2018 Jan 4;36(2):313–21. doi: 10.1016/j.vaccine.2017.10.079 (PMC5756538; doi:10.1016/j.vaccine.2017.10.079)
Supplement: Supplementary data 1 [file mmc1.pdf]

Immunogen	Included subtypes	Number of subtype sequences included
HCV GT1 (96 sequences included)	1a	48
	1b	48
HCV GT1/3 (72 sequences included)	1a	18
	1b	18
	3a	36
HCV GT1-6 (216 sequences included)	1a/1b (equal parts)	36
	2a/2b (equal parts)	36
	3a	36
	4a/4 other (equal parts)	36
	5	3*12
	6a/6 other (equal parts)	36

Supplementary Table S1: Sequence datasets for immunogen design. Three HCV immunogens were generated (GT1, GT1/3 and GT1-6). These were generated from HCV subtypes as given.

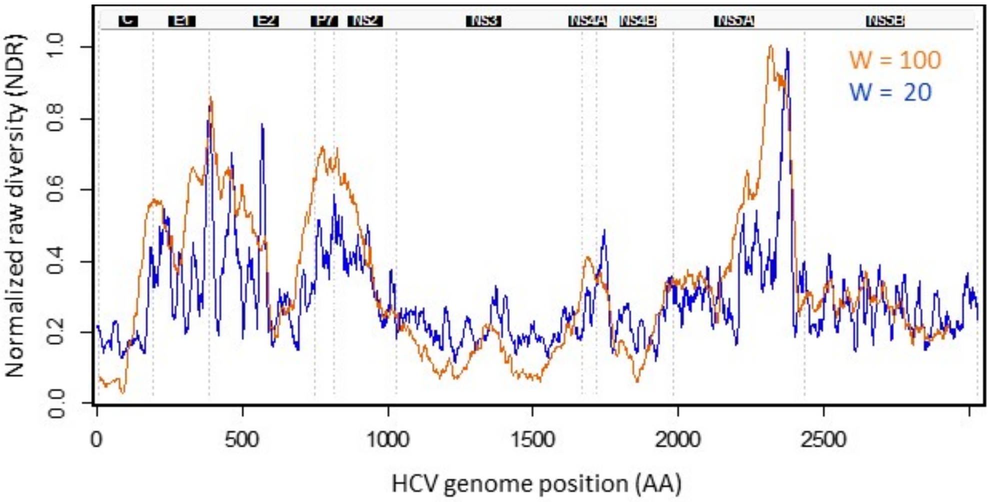

Supplementary Figure S1: Evaluation of sliding window sizes for diversity calculation. To calculate sequence diversity, a hamming distance comparison method was used. Multiple window sizes were evaluated. Pictured are window sizes W=100 and W=20. A window size of W=20 was subsequently chosen as a good compromise, possibly holding substantial variation, but still able to detect intermediate-to-long, conserved sequence segments.

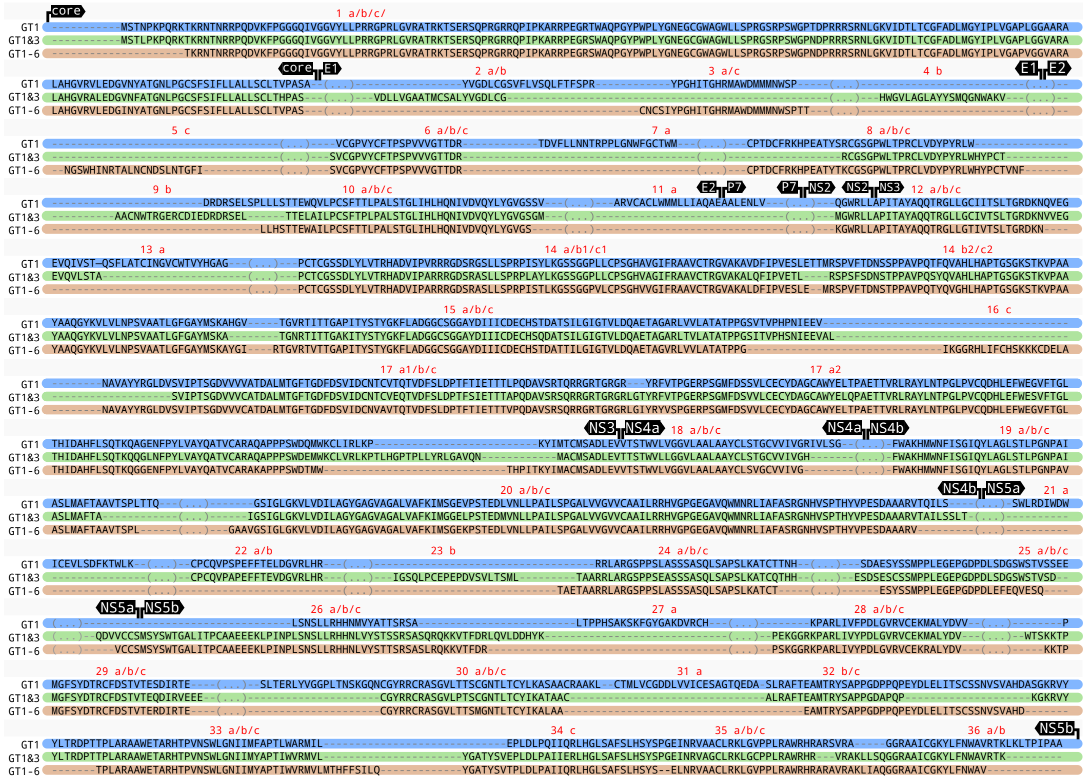

Supplementary Figure S2: Sequence overlap between conserved HCV GT1, HCV GT1/3 and HCV GT1-6 immunogens. High sequence overlap between consensus sequences of conserved segments is depicted for HCV GT1 (a, blue), HCV GT1/3 (b, green) and HCV GT1-6 (c, orange). HCV viral segments are specified, and conserved segment ID numbers are assigned according to position on the HCV genome.

Immunogen Name	Immunogen sequence
**HCV-GT1-short-TPA**	MDAMKRGLCCVLLLCGAVFVSPSQEIHARFRRkgggpgggkSTNPKPQRKTKRNTNRRPQDVKFP<br>GGGQIVGGVYLLPRRGPRLGVRATRKTSERSQPRGRRQPIPKARRPEGRTWAQPGYPWPLYGNEG<br>CGWAGWLLSPRGSRPSWGPTDPRRRSRNLGKVIDTLTCGFADLMGYIPLVGAPLGGAARALAHGV<br>RVLEDGVNYATGNLPGCSFSIFLLALLSCLTVPASAggsgDRDRSELSPLLLSTTQWQVLPCSFT<br>TLPALSTGLIHLHQNIVDVQYLYGVGSSVgpPCTCGSSDLYLVTRHADVIPVRRRGDSRGSLLSP<br>RPISYLKGSSGGPLLCPSGHAVGIFRAAVCTRGVAKAVDFIPVESMETTMRSPVFTDNSSPPAVP<br>QTFQVAHLHAPTGSGKSTKVPAAYAAQGYKVLVLNPSVAATLGFGAYMSKAHGVsgTGVRTITTG<br>SPITYSTYGKFLADGGCSGGAYDIIICDECHSTDATSILGIGTVLDQAETAGARLVVLATATPPG<br>SVTVPHPNIEEVgpgNAVAYYRGLDVSVIPTSGDVVVVATDALMTGFTGDFDSVIDCNTCVTQTV<br>DFSLDPTFTIETTTLPQDAVSRTQRRGRTGRGRpgggsgggYRFVTPGERPSGMFDSSVLCECYD<br>AGCAWYELTPAETTVRLRAYLNTPGLPVCQDHLEFWEGVFTGLTHIDAHFLSQTKQAGDNFPYLV<br>AYQATVCARAQAPPPSWDQMWKCLIRLKPggkgpggKYIMTCMSADLEVVTSTWVLVGGVLAALA<br>AYCLSTGCVVIVGRIVLSGgsgGSIGLGKVLVDILAGYGAGVAGALVAFKIMSGEVPSTEDLVNL<br>LPAILSPGALVVGVVCAAILRRHVGPGEGAVQWMNRLIAFASRGNHVSPTHYVPESDAAARVTQI<br>LSgpSLTERLYVGGPLTNSKGQNCGYRRCRASGVLTTSCGNTLTCYLKASAACRAAKLggpgSLR<br>AFTEAMTRYSAPPGDPPQPEYDLELITSCSSNVSVAHDASGKRVYYLTRDPTTPLARAAWETARH<br>TPVNSWLGNIIMYAPTLWARMILggsggEPLDLPQIIQRLHGLSAFSLHSYSPGEINRVAACLRK<br>LGVPPLRAWRHRARSVRA
**HCV-GT1-long-TPA**	MDAMKRGLCCVLLLCGAVFVSPSQEIHARFRRkgggpgggkSTNPKPQRKTKRNTNRRPQDVKFP<br>GGGQIVGGVYLLPRRGPRLGVRATRKTSERSQPRGRRQPIPKARRPEGRTWAQPGYPWPLYGNEG<br>CGWAGWLLSPRGSRPSWGPTDPRRRSRNLGKVIDTLTCGFADLMGYIPLVGAPLGGAARALAHGV<br>RVLEDGVNYATGNLPGCSFSIFLLALLSCLTVPASAggsgYVGDLCGSVFLVSQLFTFSPRpYPG<br>HITGHRMAWDMMMNWSPVCGPVYCFTPSPVVVGTTDRTDVFLLNNTRPPLGNWFGCTWMCPTDCF<br>RKHPEATYSRCGSGPWLTPRCLVDYPYRLWgDRDRSELSPLLLSTTQWQVLPCSFTTLPALSTGL<br>IHLHQNIVDVQYLYGVGSSVARVCACLWMMLLIAQAEAALENLVKGWRLLAPITAYAQQTRGLLG<br>CIITSLTGRDKNQVEGEVQIVSTQSFLATCINGVCWTVYHGAGPCTCGSSDLYLVTRHADVIPVR<br>RRGDSRGSLLSPRPISYLKGSSGGPLLCPSGHAVGIFRAAVCTRGVAKAVDFIPVESMETTMRSP<br>VFTDNSSPPAVPQTFQVAHLHAPTGSGKSTKVPAAYAAQGYKVLVLNPSVAATLGFGAYMSKAHG<br>VsgTGVRTITTGSPITYSTYGKFLADGGCSGGAYDIIICDECHSTDATSILGIGTVLDQAETAGA<br>RLVVLATATPPGSVTVPHPNIEEVgpgNAVAYYRGLDVSVIPTSGDVVVVATDALMTGFTGDFDS<br>VIDCNTCVTQTVDFSLDPTFTIETTTLPQDAVSRTQRRGRTGRGRpgggsgggYRFVTPGERPSG<br>MFDSSVLCECYDAGCAWYELTPAETTVRLRAYLNTPGLPVCQDHLEFWEGVFTGLTHIDAHFLSQ<br>TKQAGDNFPYLVAYQATVCARAQAPPPSWDQMWKCLIRLKPggkgpggKYIMTCMSADLEVVTST<br>WVLVGGVLAALAAYCLSTGCVVIVGRIVLSGgpgFWAKHMWNFISGIQYLAGLSTLPGNPAIASL<br>MAFTASVTSPLTTQpGSIGLGKVLVDILAGYGAGVAGALVAFKIMSGEVPSTEDLVNLLPAILSP<br>GALVVGVVCAAILRRHVGPGEGAVQWMNRLIAFASRGNHVSPTHYVPESDAAARVTQILSSWLRD<br>IWDWICEVLSDFKTWLKCPCQVPSPEFFTELDGVRLHRkkgpgsgpgpRRLARGSPPSLASSSAS<br>QLSAPSLKATCTTNHSDAESYSSMPPLEGEPGDPDLSDGSWSTVSSEAgsgsLSNSLLRHHNMVY<br>ATTSRSAgpLTPPHSAKSKFGYGAKDVRCHsgsgggsKPARLIVFPDLGVRVCEKMALYDVVggP<br>MGFSYDTRCFDSTVTESDIRTEggsgggSLTERLYVGGPLTNSKGQNCGYRRCRASGVLTTSCGN<br>TLTCYLKASAACRAAKLggsggCTMLVCGDDLVVICESAGTQEDASLRAFTEAMTRYSAPPGDPP<br>QPEYDLELITSCSSNVSVAHDASGKRVYYLTRDPTTPLARAAWETARHTPVNSWLGNIIMYAPTL<br>WARMILggsggEPLDLPQIIQRLHGLSAFSLHSYSPGEINRVAACLRKLGVPPLRAWRHRARSVR<br>AsGGRAAICGKYLFNWAVRTKLKLTPIPAA
**HCV-GT1/3-short-TPA**	MDAMKRGLCCVLLLCGAVFVSPSQEIHARFRRkgggpgggkSTNPKPQRKTKRNTNRRPQDVKFP<br>GGGQIVGGVYLLPRRGPRLGVRATRKTSERSQPRGRRQPIPKARRPEGRSWAQPGYPWPLYGNEG<br>CGWAGWLLSPRGSRPSWGPTDPRRRSRNLGKVIDTLTCGFADLMGYIPLVGAPLGGAARALAHGV<br>RVLEDGVNYATGNLPGCSFSIFLLALLSCLTVPASgkggTTELAILPCSFTPLPALSTGLIHLHQ<br>NIVDVQYLYGVGSGMMGWRLLAPITAYAQQTRGLLGTIVTSLTGRDKNVVTGEVQVLSTAgsgPC<br>TCGSADLYLVTRDADVIPARRRGDSTASLLSPRPLACLKGSSGGPVMCPSGHVAGIFRAAVCTRG<br>VAKALQFIPVETLRSPVFSDNSSPPAVPQSYQVAHLHAPTGSGKSTKVPAAYAAQGYKVLVLNPS<br>VAATLGFGAYMSKATGNRTITTGAKLTYSTYGKFLADGGCSGGAYDVIICDECHAQDATSILGIG<br>TVLDQAETAGVRLTVLATATPPGSITVPHSNIEEVALSVIPTAGDVVVCATDALMTGFTGDFDSV<br>IDCNVAVEQYVDFSLDPTFSIETRTAPQDAVSRSQRRGRTGRGRLGTYRYVGPGERPSGMFDSVV<br>LCECYDAGCAWYELQPAETTVRLRAYLSTPGLPVCQDHLDFWESVFTGLTHIDAHFLSQTKQQGL<br>NFPYLTAYQATVCARAQAPPPSWDEMWKCLVRLKPTLHGPTPLLYRLGPVQNggsgkggIGSVGL<br>GKVLVDILAGYGAGVAGALVAFKIMSGEIPSTEDLVNLLPAILSPGALVVGVVCAAILRRHVGPG<br>EGAVQWMNRLIAFASRGNHVSPTHYVPESDAAARVTAILSSLTsgQSVVCCSMSYSWTGALITPC<br>SAEEEKLPINPLSNSLLRHHNLVYSTSSRSASQRQKKVTFDRLQVLDDHYKKGKRYYYLTRDPTT<br>PLARAAWETARHTPVNSWLGNIIMYAPTIWVRMVMgkgpgsYGATYSVTPLDLPAIIERLHGLSA<br>FTLHSYSPVELNRVAGTLRKLGCPPLRAWRHR
**HCV-GT1/3-long-TPA**	MDAMKRGLCCVLLLCGAVFVSPSQEIHARFRRkgggpgggkSTNPKPQRKTKRNTNRRPQDVKFP<br>GGGQIVGGVYLLPRRGPRLGVRATRKTSERSQPRGRRQPIPKARRPEGRSWAQPGYPWPLYGNEG<br>CGWAGWLLSPRGSRPSWGPTDPRRRSRNLGKVIDTLTCGFADLMGYIPLVGAPLGGAARALAHGV<br>RVLEDGVNYATGNLPGCSFSIFLLALLSCLTVPASgggsgggVDLLVGAATMCSALYVGDMCGpH<br>WGVLAGLAYYSMQGNWAKVSVCGPVYCFTPSPVVVGTTDRgpgsgkgpggRCGSGPWLTPRCLVD<br>YPYRLWHYPCTAACNWTRGERCDIEDRDRSELggsgTTELAILPCSFTPLPALSTGLIHLHQNIV<br>DVQYLYGVGSGMMGWRLLAPITAYAQQTRGLLGTIVTSLTGRDKNVVTGEVQVLSTAgsgPCTCG<br>SADLYLVTRDADVIPARRRGDSTASLLSPRPLACLKGSSGGPVMCPSGHVAGIFRAAVCTRGVAK<br>ALQFIPVETLRSPVFSDNSSPPAVPQSYQVAHLHAPTGSGKSTKVPAAYAAQGYKVLVLNPSVAA<br>TLGFGAYMSKATGNRTITTGAKLTYSTYGKFLADGGCSGGAYDVIICDECHAQDATSILGIGTVL<br>DQAETAGVRLTVLATATPPGSITVPHSNIEEVALSVIPTAGDVVVCATDALMTGFTGDFDSVIDC<br>NVAVEQYVDFSLDPTFSIETRTAPQDAVSRSQRRGRTGRGRLGTYRYVGPGERPSGMFDSVVLCE<br>CYDAGCAWYELQPAETTVRLRAYLSTPGLPVCQDHLDFWESVFTGLTHIDAHFLSQTKQQGLNFP<br>YLTAYQATVCARAQAPPPSWDEMWKCLVRLKPTLHGPTPLLYRLGPVQNgppMACMSADLEVTTS<br>TWVLLGGVLAALAAYCLSVGCVVIVGHFWAKHMWNFISGIQYLAGLSTLPGNPAIASLMAFTAgp<br>IGSVGLGKVLVDILAGYGAGVAGALVAFKIMSGEIPSTEDLVNLLPAILSPGALVVGVVCAAILR<br>RHVGPGEGAVQWMNRLIAFASRGNHVSPTHYVPESDAAARVTAILSSLTCPCQVPAPEFFTEVDG<br>VRLHRgggppgggIGSQLPCEPEPDVSVLTSMLpTAARRLARGSPPSEASSSASQLSAPSLKATC<br>QTHRESDSESCSSMPPLEGEPGDPDLSCDSWSTVSDQSVVCCSMSYSWTGALITPCSAEEEKLPI<br>NPLSNSLLRHHNLVYSTSSRSASQRQKKVTFDRLQVLDDHYKgpgPEKGGRKPARLIVYPDLGVR<br>VCEKMALYDVpgpgggWTSKKTPMGFSYDTRCFDSTVTEQDIRVEEEgpCGYRRCRASGVLTTSC<br>GNTLTCYIKARAACggsggALRAFTEAMTRYSAPPGDAPQPggKGKRYYYLTRDPTTPLARAAWE<br>TARHTPVNSWLGNIIMYAPTIWVRMVMgkgpgsYGATYSVTPLDLPAIIERLHGLSAFTLHSYSP<br>VELNRVAGTLRKLGCPPLRAWRHRggpggpggVRAKLLSQGGRAAICGKYLFNWAVRTK

**Immunogen Name**	**Immunogen sequence**
**HCV-GT1-6-short-TPA**	MDAMKRGLCCVLLLCGAVFVSPSQEIHARFRRkgggpgggTKRNTNRRPMDVKFPGGGQIVGGVY<br>LLPRRGPRLGVRATRKTSERSQPRGRRQPIPKARRPEGRSWAQPGYPWPLYGNEGCGWAGWLLSP<br>RGSRPSWGPNDPRRRSRNLGKVIDTLTCGFADLMGYIPLVGAPVGGVARALAHGVRALEDGINYA<br>TGNLPGCSFSIFLLALLSCLTVPASCPTDCFRKHPEATYTKCGSGPWLTPRCLVDYPYRLWHYPC<br>TVNFgsgLLLSTTEWQILPCSFTTLPALSTGLIHLHQNIVDVQYLYGVGSgpPCTCGSSDLYLVT<br>RHADVIPVRRRGDSRGSLLSPRPISYLKGSSGGPVLCPSGHAVGIFRAAVCTRGVAKAVDFIPVE<br>SLEMRSPVFTDNSTPPAVPQTYQVAHLHAPTGSGKSTKVPAAYAAQGYKVLVLNPSVAATLGFGA<br>YMSKAYGIggsRSGVRTITTGAPITYSTYGKFLADGGCSGGAYDIIICDECHSTDSTTILGIGTV<br>LDQAETAGVRLVVLATATPPGgNAVAYYRGLDVSVIPTSGDVVVVATDALMTGFTGDFDSVIDCN<br>TCVTQTVDFSLDPTFTIETTTVPQDAVSRSQRRGRTGRGRRGIYRFVTPGERPSGMFDSSVLCEC<br>YDAGCAWYELTPAETSVRLRAYLNTPGLPVCQDHLEFWEGVFTGLTHIDAHFLSQTKQAGDNFPY<br>LVAYQATVCARAQAPPPSWDQMWTHPITKYIMACMSADLEVVTSTWVLVGGVLAALAAYCLSVGS<br>VVIVGgpgFWAKHMWNFISGIQYLAGLSTLPGNPAIASLMAFTAAVTSPLGAAVGSVGLGKVLVD<br>ILAGYGAGVAGALVAFKIMSGEVPSTEDLVNLLPAILSPGALVVGVVCAAILRRHVGPGEGAVQW<br>MNRLIAFASRGNHVSPTHYVPESDAAARVgggsgggVCCSMSYSWTGALITPCAAEEEKLPINPL<br>SNSLIRHHNMVYSTTSRSASLRQKKVTFDRgggkgggpTPLARAAWETARHTPVNSWLGNIIMYA<br>PTIWVRMVLMTHFFSILQgggsggpELNRVGACLRKLGVPPLRAWRHRARAVRAKLIAQGGKAAI<br>CGKYLFNWAV
**HCV-GT1-6-long-TPA**	MDAMKRGLCCVLLLCGAVFVSPSQEIHARFRRkgggpgggTKRNTNRRPMDVKFPGGGQIVGGVY<br>LLPRRGPRLGVRATRKTSERSQPRGRRQPIPKARRPEGRSWAQPGYPWPLYGNEGCGWAGWLLSP<br>RGSRPSWGPNDPRRRSRNLGKVIDTLTCGFADLMGYIPLVGAPVGGVARALAHGVRALEDGINYA<br>TGNLPGCSFSIFLLALLSCLTVPASpgCNCSIYPGHITGHRMAWDMMMNWSPTTkkNGSWHINRT<br>ALNCNDSLNTGFIgpggSVCGPVYCFTPSPVVVGTTDRgpgCPTDCFRKHPEATYTKCGSGPWLT<br>PRCLVDYPYRLWHYPCTVNFgsgLLLSTTEWQILPCSFTTLPALSTGLIHLHQNIVDVQYLYGVG<br>SMGWRLLAPITAYAQQTRGLLGTIVTSLTGRDKNPCTCGSSDLYLVTRHADVIPVRRRGDSRGSL<br>LSPRPISYLKGSSGGPVLCPSGHAVGIFRAAVCTRGVAKAVDFIPVESLEMRSPVFTDNSTPPAV<br>PQTYQVAHLHAPTGSGKSTKVPAAYAAQGYKVLVLNPSVAATLGFGAYMSKAYGIggsRSGVRTI<br>TTGAPITYSTYGKFLADGGCSGGAYDIIICDECHSTDSTTILGIGTVLDQAETAGVRLVVLATAT<br>PPGgkggkgIKGGRHLIFCHSKKKCDELAgpgNAVAYYRGLDVSVIPTSGDVVVVATDALMTGFT<br>GDFDSVIDCNTCVTQTVDFSLDPTFTIETTTVPQDAVSRSQRRGRTGRGRRGIYRFVTPGERPSG<br>MFDSSVLCECYDAGCAWYELTPAETSVRLRAYLNTPGLPVCQDHLEFWEGVFTGLTHIDAHFLSQ<br>TKQAGDNFPYLVAYQATVCARAQAPPPSWDQMWTHPITKYIMACMSADLEVVTSTWVLVGGVLAA<br>LAAYCLSVGSVVIVGgpgFWAKHMWNFISGIQYLAGLSTLPGNPAIASLMAFTAAVTSPLGAAVG<br>SVGLGKVLVDILAGYGAGVAGALVAFKIMSGEVPSTEDLVNLLPAILSPGALVVGVVCAAILRRH<br>VGPGEGAVQWMNRLIAFASRGNHVSPTHYVPESDAAARVgpTAETAARRLARGSPPSLASSSASQ<br>LSAPSLKATCTVCCSMSYSWTGALITPCAAEEEKLPINPLSNSLIRHHNMVYSTTSRSASLRQKK<br>VTFDRggsggpgPSKGGRKPARLIVYPDLGVRVCEKRALYDVpggpKKTPMGFSYDTRCFDSTVT<br>ERDIRTEgggpggCGYRRCRASGVLTTSMGNTITCYIKALAAEAMTRYSAPPGDPPQPEYDLELI<br>TSCSSNVSVAHDggsggTPLARAAWETARHTPVNSWLGNIIMYAPTIWVRMVLMTHFFSILQggp<br>gYGATYSVTPLDLPAIIERLHGLSAFTLHSYSggpggpELNRVGACLRKLGVPPLRAWRHRARAV<br>RAKLIAQGGKAAICGKYLFNWAV
**HCV-GT1-6-long-TPA-nolinkers**	MDAMKRGLCCVLLLCGAVFVSPSQEIHARFRRkgggpgggTKRNTNRRPMDVKFPGGGQIVGGVY<br>LLPRRGPRLGVRATRKTSERSQPRGRRQPIPKARRPEGRSWAQPGYPWPLYGNEGCGWAGWLLSP<br>RGSRPSWGPNDPRRRSRNLGKVIDTLTCGFADLMGYIPLVGAPVGGVARALAHGVRALEDGINYA<br>TGNLPGCSFSIFLLALLSCLTVPASCNCSIYPGHITGHRMAWDMMMNWSPTTNGSWHINRTALNC<br>NDSLNTGFISVCGPVYCFTPSPVVVGTTDRCPTDCFRKHPEATYTKCGSGPWLTPRCLVDYPYRL<br>WHYPCTVNFLLLSTTEWQILPCSFTTLPALSTGLIHLHQNIVDVQYLYGVGSMGWRLLAPITAYA<br>QQTRGLLGTIVTSLTGRDKNPCTCGSSDLYLVTRHADVIPVRRRGDSRGSLLSPRPISYLKGSSG<br>GPVLCPSGHAVGIFRAAVCTRGVAKAVDFIPVESLEMRSPVFTDNSTPPAVPQTYQVAHLHAPTG<br>SGKSTKVPAAYAAQGYKVLVLNPSVAATLGFGAYMSKAYGIRSGVRTITTGAPITYSTYGKFLAD<br>GGCSGGAYDIIICDECHSTDSTTILGIGTVLDQAETAGVRLVVLATATPPGIKGGRHLIFCHSKK<br>KCDELANAVAYYRGLDVSVIPTSGDVVVVATDALMTGFTGDFDSVIDCNTCVTQTVDFSLDPTFT<br>IETTTVPQDAVSRSQRRGRTGRGRRGIYRFVTPGERPSGMFDSSVLCECYDAGCAWYELTPAETS<br>VRLRAYLNTPGLPVCQDHLEFWEGVFTGLTHIDAHFLSQTKQAGDNFPYLVAYQATVCARAQAPP<br>PSWDQMWTHPITKYIMACMSADLEVVTSTWVLVGGVLAALAAYCLSVGSVVIVGFWAKHMWNFIS<br>GIQYLAGLSTLPGNPAIASLMAFTAAVTSPLGAAVGSVGLGKVLVDILAGYGAGVAGALVAFKIM<br>SGEVPSTEDLVNLLPAILSPGALVVGVVCAAILRRHVGPGEGAVQWMNRLIAFASRGNHVSPTHY<br>VPESDAAARVTAETAARRLARGSPPSLASSSASQLSAPSLKATCTVCCSMSYSWTGALITPCAAE<br>EEKLPINPLSNSLIRHHNMVYSTTSRSASLRQKKVTFDRPSKGGRKPARLIVYPDLGVRVCEKRA<br>LYDVKKTPMGFSYDTRCFDSTVTERDIRTECGYRRCRASGVLTTSMGNTITCYIKALAAEAMTRY<br>SAPPGDPPQPEYDLELITSCSSNVSVAHDTPLARAAWETARHTPVNSWLGNIIMYAPTIWVRMVL<br>MTHFFSILQYGATYSVTPLDLPAIIERLHGLSAFTLHSYSELNRVGACLRKLGVPPLRAWRHRAR<br>AVRAKLIAQGGKAAICGKYLFNWAV

Supplementary Table S2: Name and immunogen sequence for seven defined conserved immunogens are specified. TPA leader sequence (TPA-LS, underlined), and linker regions between conserved regions to abrogate artificial epitopes (lower case) are depicted.

Immunogen name	Length of immunogen (AA)	Number of cleavage sites
GT1-short-TPA	1123	368
GT1-long-TPA	1655	545
GT1/3-short-TPA	1072	357
GT1/3-long-TPA	1554	506
GT1-6-short-TPA	1115	374
GT1-6-long-TPA	1453	487
GT1-6-long-TPA-nolinkers	1390	462

Supplementary Table S3: Number of predicted proteasomal cleavage sites. The total length of each conserved immunogen and the number of proteasomal cleavage sites predicted by NetChop 3.1 Server (version C-term, http://www.cbs.dtu.dk/services/NetChop/), respectively. AA amino acids.

Immunogen name	Number of strong binding epitopes predicted (5-20AA)	Number of epitopes falling in junction regions
GT1-short-TPA	**19**	**5**
GT1-long-TPA	**23**	**9**
GT1/3-short-TPA	**18**	**4**
GT1/3-long-TPA	**23**	**10**
GT1-6-short-TPA	**17**	**4**
GT1-6-long-TPA	**18**	**6**
GT1-6-long-TPA-nolinkers		

Supplementary Table S4: B-cell prediction analysis assessing potential immunogenicity of conserved HCV immunogens. B-cell epitopes were predicted with BepiPred 2.0 using a cut-off for strong binding epitopes 0.55 and an epitope prediction length of 5 to 20 AAs. AA Amino acids.

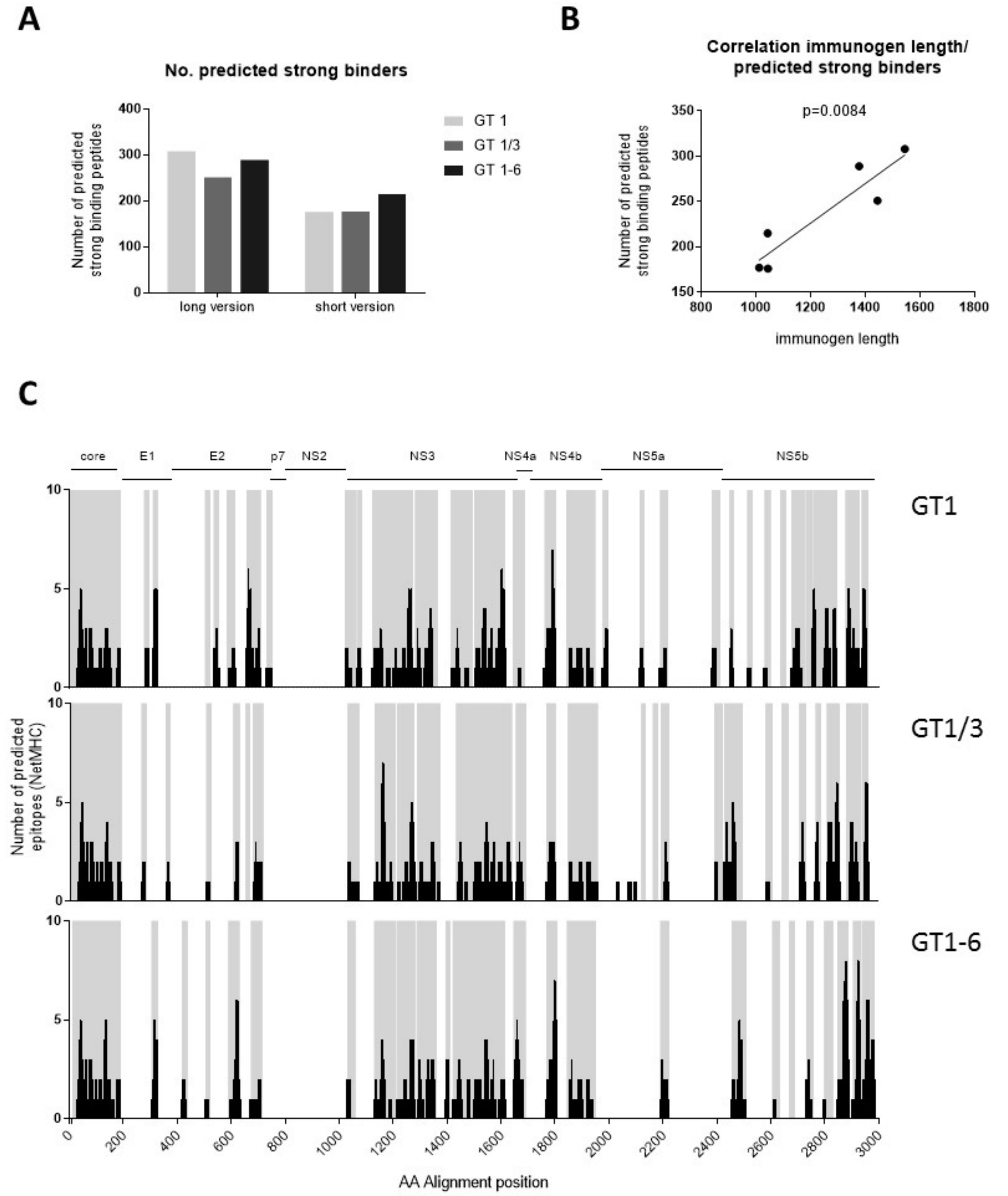

Supplementary Figure S3: T-cell epitope prediction analysis assessing potential immunogenicity of conserved HCV immunogens. (A) Number of total strong binders predicted using the NetMHC online epitope prediction algorithm (http://www.cbs.dtu.dk/services) to define strong binding HLA class-I epitopes for short and long versions of conserved HCV GT1, GT1/3 and GT1-6 immunogens. (B) Immunogen length directly correlates with numbers of strong binders predicted (p=0.0084) (C) Depiction of predicted strong binding epitopes distributed across a map of the HCV genome. Conserved sequence segments defined within HCV GT1 (top), GT1/3 (middle) and GT1-6 (bottom) are marked in grey. Epitopes in junction areas and epitope duplicates with identical HLA types, but differing predicted epitope lengths are not depicted.

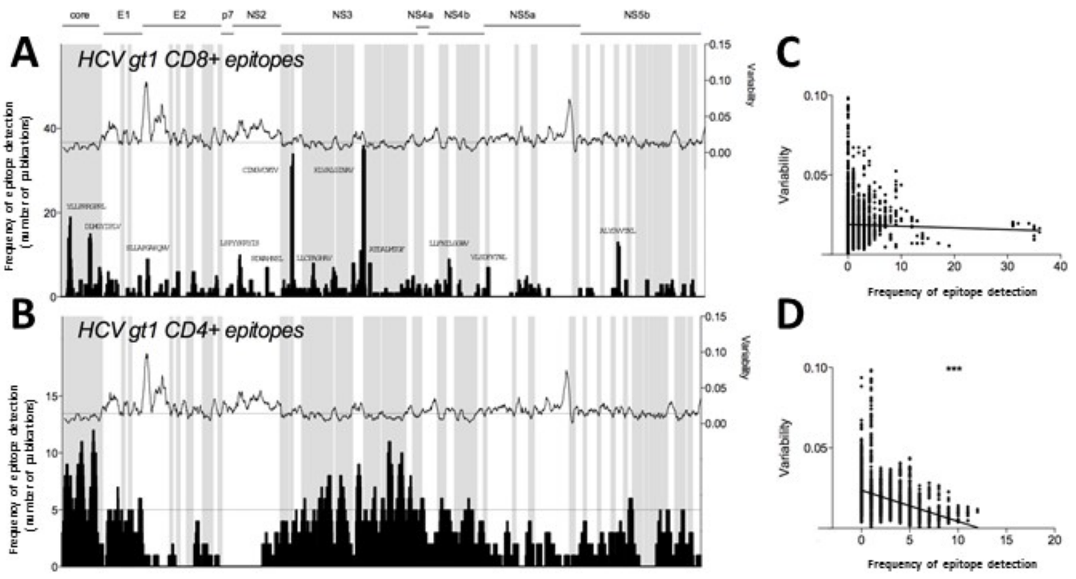

Supplementary Figure S4: Epitopes defined in natural HCV infection populate HCV conserved and variable regions. Plot of HCV GT1 sequence heterogeneity with (A) HCV genotype-1 CD8+ and (B) CD4+ epitopes described in natural HCV genotype-1 infection. Right y-axis: HCV genotype-1 sequence variability is depicted with conserved segments marked in grey (below dashed line). HCV viral regions are marked at the top. Left Y-axis: Frequency of epitopes detection in natural HCV infection reported on the Immune Epitope Database (IEDB). The number of publications in which HCV genotype-1 specific epitopes were described is depicted with the epitope position. Duplicates of CD8+ epitopes were excluded if epitopes had matching HLA types, as well as CD4+ epitopes where described sequences differed by less than 2 amino acids. For selected immunodominant epitopes sequence information is shown. (C+D) Correlation between sequence conservation and epitope detection frequency is shown for CD8+ (C) and CD4+ (D) epitopes (as assessed by number of publications in which an epitope was detected at each amino acid position). CD4+ epitopes were frequently detected at conserved amino acid positions ($p<0.0001$), whereas CD8+ epitopes were detected at both conserved and variable amino acid positions.

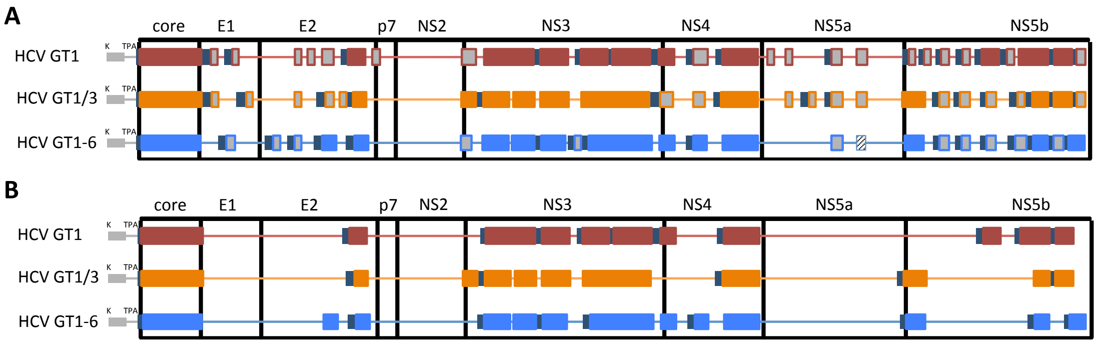

Supplementary Figure S5: Linker insertion into long and short HCV conserved immunogens. (A) Long and (B) short version of conserved HCV immunogens for HCV GT1 (red), GT1/3 (orange) and GT1-6 (blue) including designed linkers marked in dark blue are depicted. K Kozak sequence. TPA Tissue plasminogen activator.

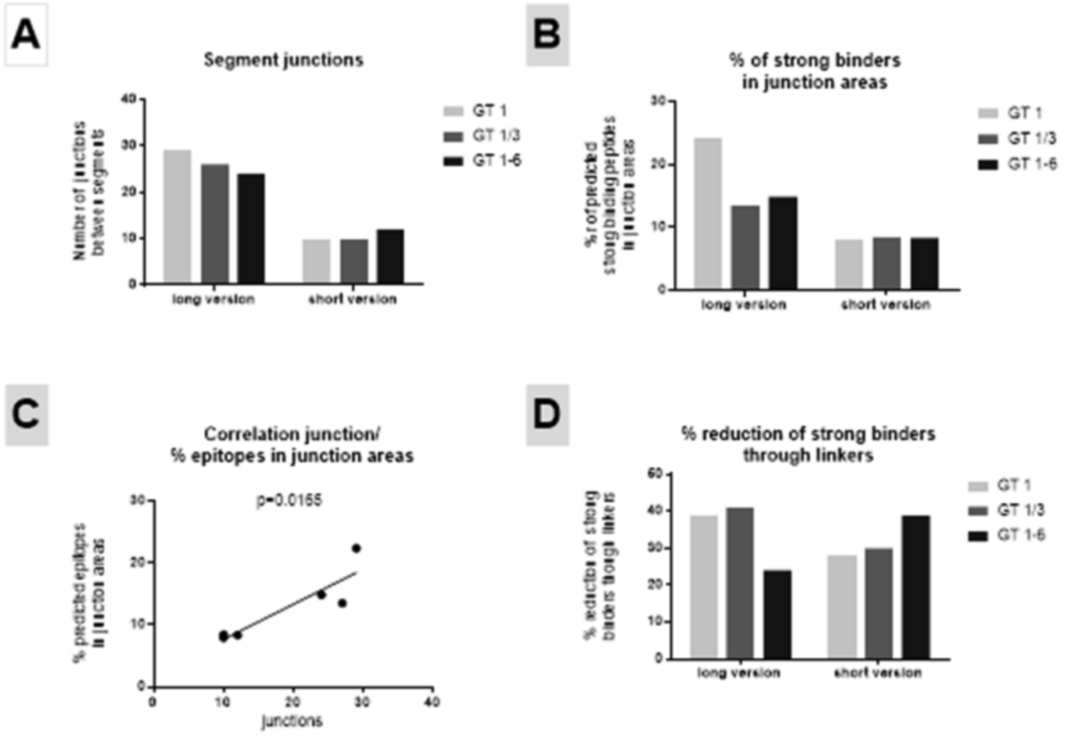

Supplementary Figure S6: Abrogation of artificial, non-HCV epitopes predicted in junction regions between concatenated conserved sequence segments. (A) The number of segment junctions between concatenated conserved HCV sequence segments is depicted for long and short immunogens. (B) The percentage of strong binding epitopes that fall onto junctional regions is shown. (C) The number of predicted epitopes in junctional regions is directly correlated with number of segment junctions between conserved segments. (D) Design of peptide linkers consisting of glycine/proline or glycine/serine combinations abrogates predicted strong binding epitopes in junctional regions.
